# Distribution characteristics of microbial community structure in atmospheric particulates of the typical industrial city in Jiangsu province, China

**DOI:** 10.1080/21655979.2021.1885223

**Published:** 2021-02-10

**Authors:** Xingcheng Yuan, Guangchao Li, Weihua Yang, Dan Li

**Affiliations:** School of Chemistry and Materials Science, Jiangsu Key Laboratory of Green Synthetic Chemistry for Functional Materials, Jiangsu Normal University, Xuzhou, P.R. China

**Keywords:** Atmospheric particulates, microbial community, industrial city, haze, PM_2.5_, PM_10_, Xuzhou

## Abstract

In this study, Xuzhou, a typical industrial city in the north of Jiangsu Province, was chosen to investigate the pollution level of atmospheric particulates. The proportion of fine particles (PM_2.5_) in PM_10_ is larger than that of coarse particles (about 58%). The physicochemical properties of PM_2.5_ were analyzed by SEM and EDS. DGGE was used to study the distribution characteristics of bacterial community structure on atmospheric particulates (TSP, PM_2.5_ and PM_10_) in different functional areas of Xuzhou city during the winter haze. It was found that the microbial populations of atmospheric particles were mainly divided into three groups: *Proteobacteria, Bacteroidetes, and Pachytenella*. The community structure of bacteria in fine particle size was more abundant than that in coarse particle size. When haze occurs, the concentration of all kinds of pathogens in fine particle size will increase. Therefore, it is necessary to focus on the monitoring and management of fine particles.

## GRAPHICAL ABSTRACT

**Figure:**
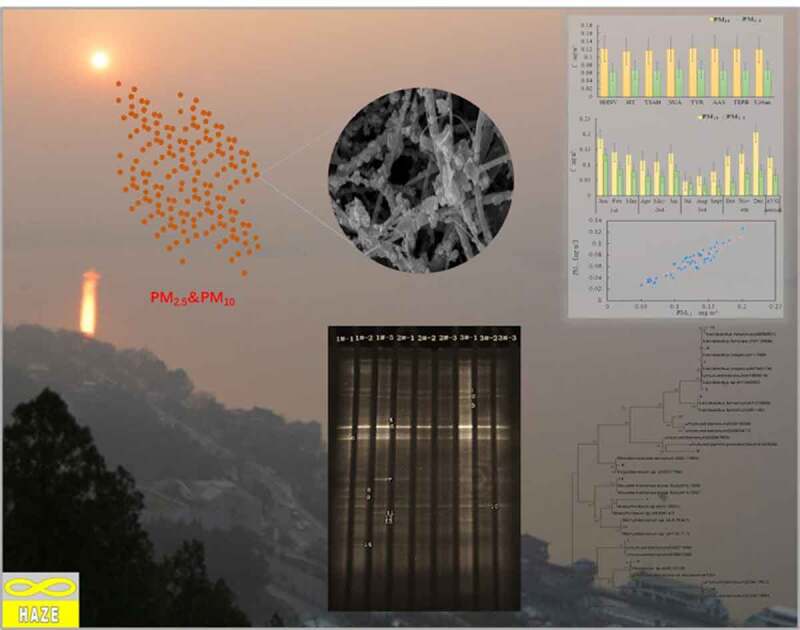

## Introduction

In recent years, most cities frequently have severe haze weather in China, and atmospheric particles are an important factor to cause haze weather [1,2]. The source, composition, migration, and transformation of particulates (total suspended particle, TSP and particulate matter, PM_10_ and PM_2.5_) in the air have attracted extensive attention [3–7]. Many of them focus on the physical and chemical properties of atmospheric particles, but little is known about the composition and characteristics of microorganisms in atmospheric particles. Therefore, it is necessary to carry out research on the source, composition, population structure, and distribution characteristics of microorganisms in atmospheric particulates.

Microorganisms in the air mainly exist in the form of microbial aerosols, including living organisms such as air bacteria, fungi, and actinomycetes [8]. It is attached to the dust, particles, and aerosols suspended in the air, a large part of which are pathogens. It can be widely spread with the flow of air, and it can damage human beings, animals, and plants under appropriate conditions, resulting in the occurrence and spread of diseases. In addition, air microorganism is an integral part of the ecosystem in the corresponding area, which can spread and transfer with the flow of aerosols in the atmosphere, and then change the microbial community structure in the air in the corresponding area. Especially in recent years, it has been gradually recognized that the fine particles PM_10_ and PM_2.5_ in aerosols are easy to accumulate bacteria and viruses in the air, and can enter human lungs, causing serious harm to human health, and many respiratory diseases are also transmitted through microbial aerosols [9–11, 12, 30].

However, there is no official exposure level and relevant regulations for air microorganism at home and abroad [13]. At present, neither EPA (U.S. Environmental Protection Agency) nor NIOSH (National Institute for Occupational Safety and Health) have proposed the microbial concentration limit. The ecological research center of Chinese Academy of Sciences has formulated the air microorganism standard, but it is only limited to a reference index of the total number of bacteria.

In the United States, France, Sweden, and other developed countries, as well as Beijing, Shanghai and other places in China, the microorganism in the air has been investigated [14–18]. Among them, the most common method depends on microbial culture to detect air microorganisms [19–21]. Previous culture experiments have shown that *Bacillus, Clavibacter, Corynebacterium, Curtobacterium, Micrococcus, Pseudomonas* and *Staphylococcus* are common bacteria in air microorganism. Because many microorganisms in nature are not culturable, the pure culture of microorganisms cannot fully reflect all microbial groups in the air, and the culture medium used in the pure culture method has obvious selectivity for microbial groups, and cannot really reflect the original microbial group’s population structure and changes. In addition, the cultivation and identification of bacteria need a long time and a lot of manpower, especially in the long-term ecological investigation with a large number of samples. Compared with pure culture, molecular ecology can overcome the above shortcomings. For example, molecular ecological methods such as clone library construction, DNA fingerprint analysis, real-time quantitative PCR, and gene chip have been used in the research of air microorganism [16,22–25]. However, there are few reports about using molecular ecology to study air microorganism in China.

Therefore, Xuzhou, a typical industrial city in northern Jiangsu Province, was selected to investigate the pollution level of atmospheric particulate matter. The community structure was analyzed by molecular ecology method. The relationship between microorganism and environmental factors was explored through community distribution. More comprehensive investigation of particulate matter pollution can provide technical support for environmental management in this area.

## Materials and methods

### Sampling methods

Seven sampling points were selected in Xuzhou City, namely, Huanghe New Village (HHNV, residential area), Huaita (HT, scenic area), Tongshan Animal Hospital (TSAH, residential area), New Urban Area (NUA, mixed traffic residential area), Taoyuan road (TYR, general industrial area), Academy of Agricultural Sciences (AAS, mixed traffic residential area), Tongshan Environmental Protection Bureau (TEPB, cultural area).

PM_10_ and PM_2.5_ samples were collected on glass fiber filter membrane by intelligent integrated sampler (Laoshan 2050B, Qingdao) and PM_10_ and PM_2.5_ cutters, according to ‘Technical specifications manual methods for ambient air quality monitoring’ (HJ 194–2017). The analytical method was based on the ‘Determination of atmospheric articles PM_10_ and PM_2.5_ in ambient air by gravimetric method’ (HJ 618–2011) .

Preparation of filter membrane: before sampling, place the glass fiber filter membrane in the desiccator for 24 hours, weigh it with an analytical balance with a sensitivity of 0.1 mg, put it in the desiccator for 1 hour, and then weigh it. The difference between the two times of mass shall not be greater than 0.4 mg (i.e. constant weight).

### Microscopic observation

Scanning electron microscopy (SEM) and energy dispersive spectrometer (EDS) were employed to observe the microscopic characteristics of atmospheric articles. The preparation of electron microscope samples was to use double-sided adhesive to stick the cut sample 1 cm × 1 cm flat on the conductive metal gasket, put the sample into the electron microscope sample bin after spraying gold (Gold plating of ion sputtering instrument, E-1010, Japan HITACHI), and use the field emission environment scanning electron microscope (QUANTA-200, USA FEI) to observe the micromorphology of particles. The working voltage of electron microscope is of 10 kV, the working current of 10 μA, the diameter of electron beam spot of 1 ~ 2 μm, and the scanning working distance of 7.8 mm. The signal acquisition time of EDS(EDAX Genesis 2000, USA FEI) analysis is 100 s, making the total value exceed 100 000.

### Microbial community analysis

#### DNA extraction

The samples for microbial community analysis were collected from glass fiber filter membrane under aseptic conditions. The DNA was then extracted using the phenolic-chloroform method [26]. Briefly, the broken fibers were suspended in 5 ml of buffer solution (0.1 M Tris-HCl, 0.1 M EDTA-Na_2_, 0.2 M NaCl, 2%CTAB;pH 8.0) in a raw extract tube, and then shaken for 45 min at 37°C. Next, 0.75 mL 20% SDS was added to the tube and the samples were incubated at 65°C for 1 h .After centrifugation at 16,000 × g for 10 min, the supernatant was recovered and washed twice with phenol-chloroform-isoamyl alcohol (25:24:1; pH 8.0). The nucleic acids were then precipitated by the addition of 0.3 M NaAC (pH 5.2) and 2 volumes of absolute ethanol for 1 h at room temperature, after which they were centrifuged for 20 min at 16,000 × g. Finally, the pellets were washed twice with 70% ethanol, dried and resuspended in 50 μL of Tris–EDTA (pH 8.0).

#### PCR amplification

**Figure 1.**
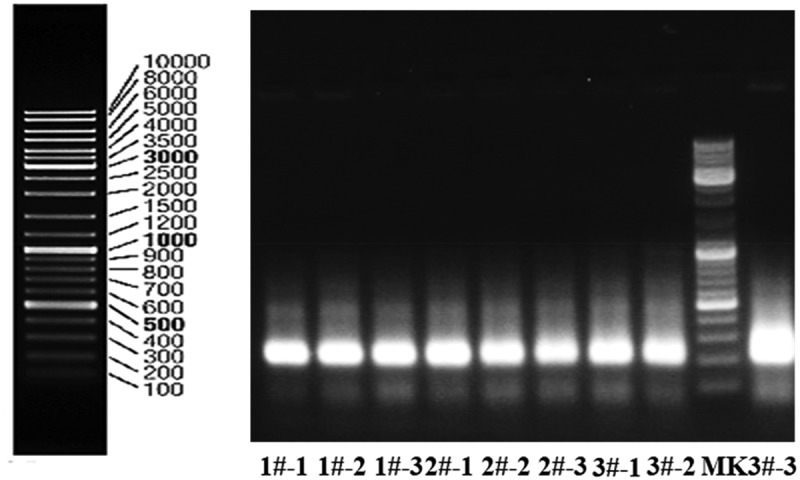
PCR amplification results

The V3 variable regions of 16S rDNA genes were amplified by PCR using the universal primers F357-GC (5ʹ-CGCCCGCCGCGCCCCGCGCCCGGCCCGCCGCCCCCGCCCCCCTACGGGAGGCAGCAG −3ʹ) and R518 (5ʹ-ATTACCGCGGCTGCTGG −3ʹ). PCR was conducted in a 50 µL reaction mixture composed of 0.5 µL DNA template, 0.25 µl of 5 U/µl Taq polymerase, 5 µL of 10× PCR buffer (2.0 mM Mg^2+^), 1 µl of 10 mM dNTP, 1 µl of each primer and 41.25 µL of ultrapure sterile water. The reaction was conducted by subjecting the samples to the following conditions: initial denaturation at 94°C for 4 min, 30 cycles of denaturation at 94°C for 0.5 min, annealing at 56°C for 1 min, and extension at 72°C for 0.5 min, and a final extension at 72°C for 7 min. The amplification results are shown in the figure 1.

### Cloning and sequencing of 16S rDNA

Cloning and sequencing of 16S rDNA was conducted by Sangon (Shanghai, China). Briefly, PCR products were purified using a PCR product purification kit, after which cloning was performed using a TA cloning kit to connect amplified fragments to pUCm-T vectors. Chemically competent E. coli cells were then transformed with the ligation mixes according to the manufacturer’s instructions and selected by blue-white selection after cultivation on Luria–Bertani (LB) agar plates containing ampicillin (100 µg/ml). The plasmid DNA was extracted using a UNIQ-10 column DNA purification kit and then sequenced with an ABI 3730 automated sequencer using the vector-specific primers M13F357 and M13R518. Each nucleotide sequence was then compared with sequences available in the GenBank database by BLAST searches. The nucleotide sequence data reported in this study were submitted to GenBank and assigned accession numbers KT000373-KT000386.

### Data processing methods

The DGGE atlas was analyzed with Quantity One software and clustered with UPGMA method [27]. The number of bands and gray value of samples were analyzed by Biodap software. Shannon diversity index, Evenness index and Simpson index were calculated according to the following formulas:

(i) Shannon diversity index (H′)

$$H{\;}^{\prime }=-\overset{S}{\underset{i=1}{\sum }}P_{i}\ln P_{i}$$

(ii) Evenness index (E)

$$E=\frac{H{\;}^{\prime }}{lnS}$$

(iii) Simpson index (D)

$$D=1-\overset{S}{\underset{i=1}{\sum }}P_{i}^{2}$$

where $P_{i}$ is the proportion of OUT (operational taxonomic units) clones to total clones, and S is the total OTU number of 16S rDNA .

The sequencing results were logged into the National Biotechnology Information Center (http://www.ncbi.nlm.nih.gov/), and the sequences were compared with the known sequences in the gen bank database. MEGA5 software and neighbor-joining method were used to construct system evolution.

## Results and discussion

In haze weather, microbes enter the human body through breathing on the attached particles. Many kinds of bacteria are important pathogenic factors of respiratory infections, skin allergies, and even asthma, lung infections, and other diseases [10, 12, 30]. The air microorganism monitoring and the result analysis are the important basis to judge the microorganism pollution level. Xuzhou is a typical industrial city with frequent haze. The atmospheric particulate matter samples were collected from seven typical urban functional areas in Xuzhou. The pollution levels of PM_10_ and PM_2.5_ were analyzed, and the physicochemical characteristics of PM_2.5_ particles were analyzed by SEM and EDS. DGGE method was used to analyze the microorganism community structure in atmospheric particulates.

### Analysis on the level of air particle pollution

The monitoring data of sampling points in Xuzhou City showed that the concentration range of PM_10_ and PM_2.5_ is 0.097–0.457 mg/m^3^ and 0.074–0.320 mg/m^3^, with the average concentration of 0.123 mg/m^3^ and 0.067 mg/m^3^ respectively. According to the ‘Ambient air quality standards’ (GB3095-2012), the exceedance of PM_10_ and PM_2.5_ in Xuzhou City is 26.3% and 31.2%. PM_10_ exceeded 85% in class I area, 28.0% in class II area, and PM_2.5_ exceeded 78.5% in class I area and 31.5% in class II area.

**Figure 2.**
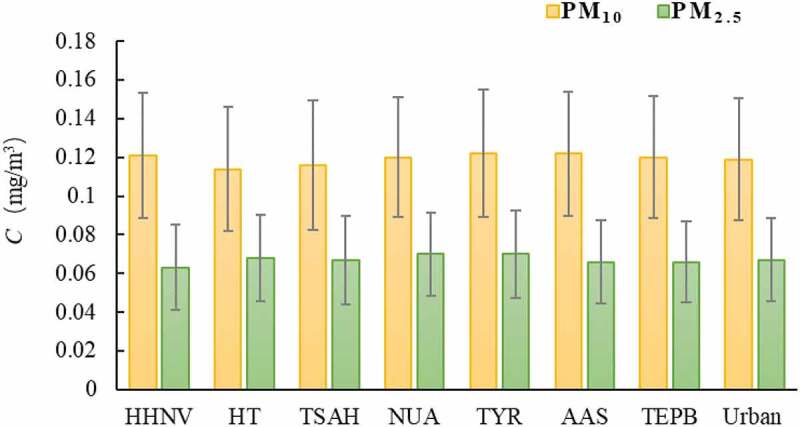
Pollution level in different functional areas

As shown in Figure 2, the pollution level of PM_10_ in different functional areas was industrial area (TYR) > mixed traffic and residential area(AAS) > cultural area (TEPB) > residential area (TSAH)> scenic area (HT). And PM_2.5_ pollution level was industrial area (TYR) = mixed traffic and residential area (AAS)> scenic area (HT) > cultural area (TEPB)> residential area (HHNV).

**Figure 3.**
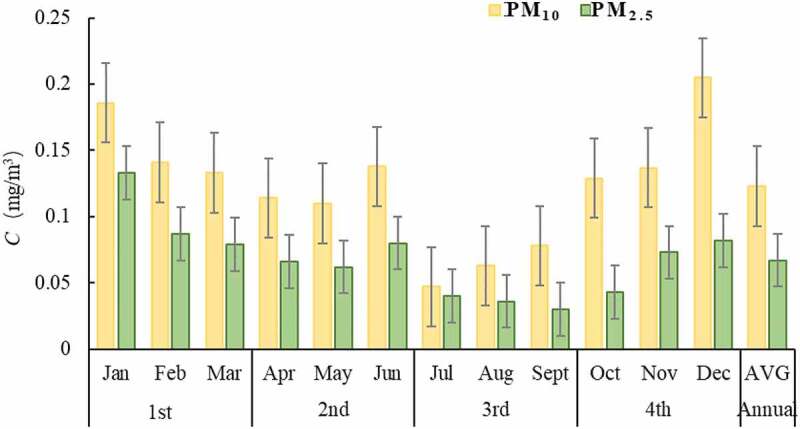
Pollution level in different months

The 12 months changes of PM_10_ and PM_2.5_ concentrations in four quarters of Xuzhou city from 2014 to 2016 are shown in Figure 3. It can be seen from Figure 3 that PM_10_ and PM_2.5_ have the same rules of seasonal change, showing the pollution change of ‘heavy in winter and light in summer’, and the seasonal difference is obvious, showing as winter > spring > autumn > summer.

There are two main reasons: on the one hand, the temperature is low, the atmosphere is stable, and the air pollutants are not easy to diffuse, and the weather is dry in winter and spring in Xuzhou, which is prone to wind and dust; on the other hand, because November, December, January and February belong to the heating season, which results in the high-quality concentration of PM_10_ and PM_2.5_ in these four months.

**Figure 4.**
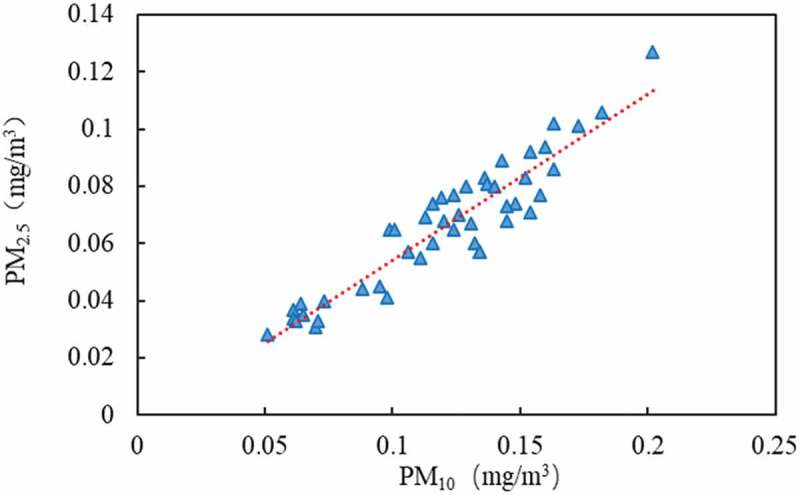
The correlation analysis of PM_2.5_ and PM_10._

PM_10_ can be divided into 2.5–10 μm coarse particles and less than 2.5 μm fine particles. Data analysis shows that the ratio of PM_2.5_/PM_10_ ranges from 0.42 to 0.66, with an average of 0.55. The correlation analysis of PM_2.5_ and PM_10_ shows that there is a significant linear relationship between them. The regression equation is PM_2.5_ = 0.581 × PM_10_-0.0037, and the correlation coefficient R^2^ = 0.8677 (n = 46). The regression analysis results are shown in Figure 4. It can be seen that the proportion of fine particles (PM_2.5_) in PM_10_ is larger than that of coarse particles, accounting for about 55%. PM_2.5_ particles are extremely small, with large specific surface area, and are easy to accumulate toxic and harmful substances in the air. With human breathing into the alveoli and blood, it is more harmful to human body [28].

### Microscopic observation of PM_2.5_

**Figure 5.**
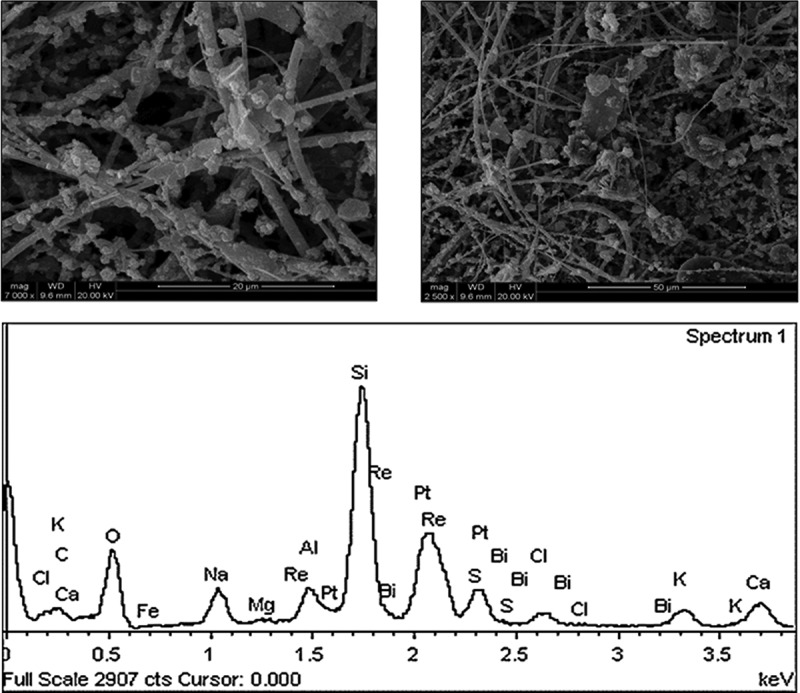
SEM and EDS of PM_2.5._

Figure 5 is SEM and EDS diagrams of PM_2.5_ sampling results, respectively. It can be seen that the particle size of most particles in the SEM picture of Figure 5 is basically below 2.5 μm, in which the larger particles in the picture have smooth surface, passivated edges and corners, and adhere to more and smaller particles, which are small, easy to handle and can float in the atmosphere for a long time. From the EDS energy spectrum, it can be seen that there are particles in the picture C, O, S, Si, Al, K, Na, Ca, Mg, Fe, and other elements can be inferred that there are a lot of mineral particles in PM_2.5_, such as quartz, calcite, clay minerals, etc.

### Distribution characteristics of microbial community structure in atmospheric particulates

#### DGGE atlas and diversity analysis

**Figure 6.**
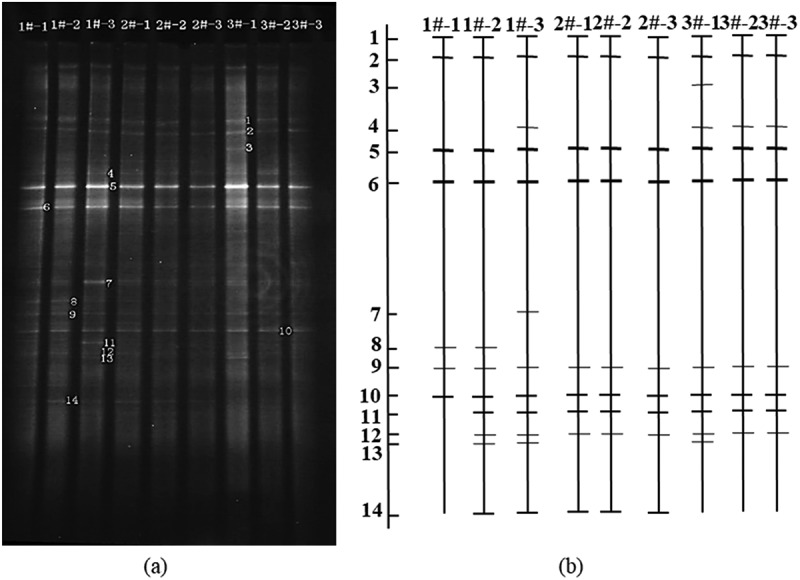
DGGE of 16S rDNA of atmospheric bacterial community

The DGGE atlas of atmospheric bacterial communities with different particle sizes at different sampling points is shown in Figure 6(a), and the distribution of each sample strip after being processed with Quantity One software is shown in Figure 6(b). Table 1 shows the diversity index of different samples.

**Table 1.** The diversity index of different samples

Sampling points	Particle size	Lane number	Band number	Shannon index	Simpson index	Evenness index
HHNV	TSP	1#-1	7	1.74	0.18	0.97
	PM_10_	1#-2	11	2.36	0.10	0.99
	PM_2.5_	1#-3	13	2.42	0.094	0.98
HT	TSP	2#-1	9	1.93	0.11	0.97
	PM_10_	2#-2	9	1.95	0.11	0.97
	PM_2.5_	2#-3	9	1.95	0.11	0.97
TSAH	TSP	3#-1	11	2.36	0.097	0.99
	PM_10_	3#-2	10	2.04	0.133	0.98
	PM_2.5_	3#-3	10	2.04	0.133	0.98

1#, 2#, and 3#, respectively, represent the sampling points of HHNV, HT, and TSAH. From No.1 to No.3, the particle size of collected air microorganism decreased gradually, which were TSP, PM_10_ and PM_2.5_ respectively. The DGGE map produced 14 recognizable bands with Shannon index between 1.74 and 2.42, indicating that the diversity of bacterial community in the atmosphere was lower than that in water, soil, and other environments.

In general, the number of bands on TSP is less, Shannon index is lower, the number of bands on PM_10_ and PM_2.5_ is increased, Shannon index is increased, indicating that the community structure of atmospheric bacteria on fine particles is more complex. Band 1, band 2, band 5, band 6 and band 10 exist in all samples and their brightness changes little, which shows that they are widely distributed in various particle sizes of air quality and have strong adaptability to the environment. Band 7 was only found in PM_2.5_ samples from HHNV, which could adapt to the specific bacteria on the fine particles in the haze weather. The brightness of band 5 and band 6 is strong, which shows that they can adapt to the change of environment and grow into the dominant bacteria in this period. The uniformity of the samples is high (the average value is >0.97), so there is little difference between the samples.

### Similarity analysis of microbial community structure

**Figure 7.**
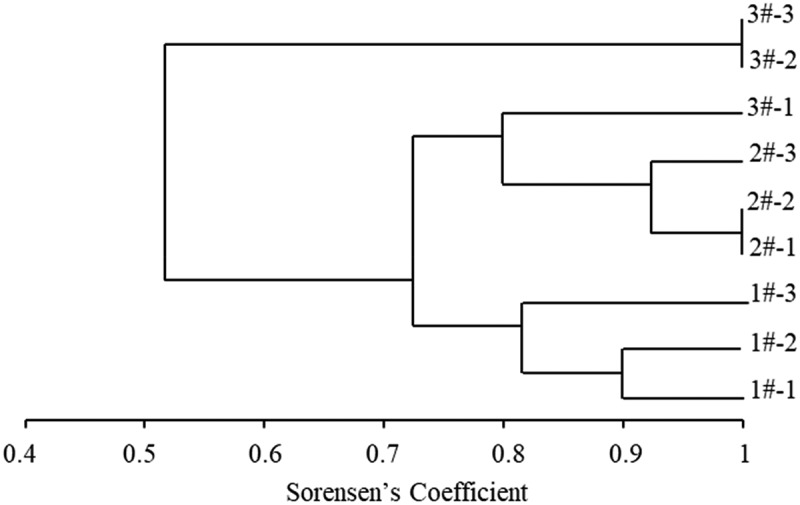
UPGMA cluster analysis chart

The unweighted group average (UPGMA) cluster analysis was carried out for different samples (Figure 7). The total is divided into two, 3#-2 and 3#-3, the similarity with other samples is 53%.The similarity between other samples is more than 70%. In addition to TSAH, the similarity of samples with different particle sizes is higher (>80%), that of PM_10_ and PM_2.5_ is higher (>90%), and that of other samples is lower.

### Clonal sequencing and phylogenetic analysis

14 bands were sequenced and analyzed, respectively. The sequencing results were compared with the existing sequences in GenBank database (Table 2), and the sequences were submitted to GenBank database (http://www.ncbi.nlm.nih.gov), and the serial number KT000373-KT000386 was obtained.

**Table 2.** BLAST comparison of DGGE sequencing bands

Phylum	Band	Closest strain	Serial number	Similarity (%)	Sequence length(bp)
*Proteobacteria*	Band2	*Bradyrhizobium sp.*	AF508147	87	171
	Band10	*Brucella melitensis biovar Suis*	AY513506	100	169
	Band11	*Methylobacterium sp. SY-2*	AJ278347	100	169
*Bacteroidetes*	Band4	*uncultured bacterium*	KF064961	97	194
	Band6	*Flexibacter sp.*	AB272379	84	188
	Band7	*uncultured bacterium*	AM697309	100	189
	Band14	*uncultured bacterium*	GQ477860	96	189
*Firmicutes*	Band1	*Lactobacillus fornicalis*	Y18654	100	194
	Band13	*Lactobacillus helveticus*	JQ805621	96	193
	Band12	*uncultured bacterium*	GQ019259	90	194
	Band3	*Lactobacillus fermentum*	X61142	100	193
	Band5	*Lactobacillus crispatus*	AF243174	95	194
*Actinobacteria*	Band9	*Microbacteriaceae bacterium Tibet-IT11*	DQ177485	97	174
*Candidatus Saccharibacteria*	Band8	*uncultured bacterium*	GQ047882	68	169

**Figure 8.**
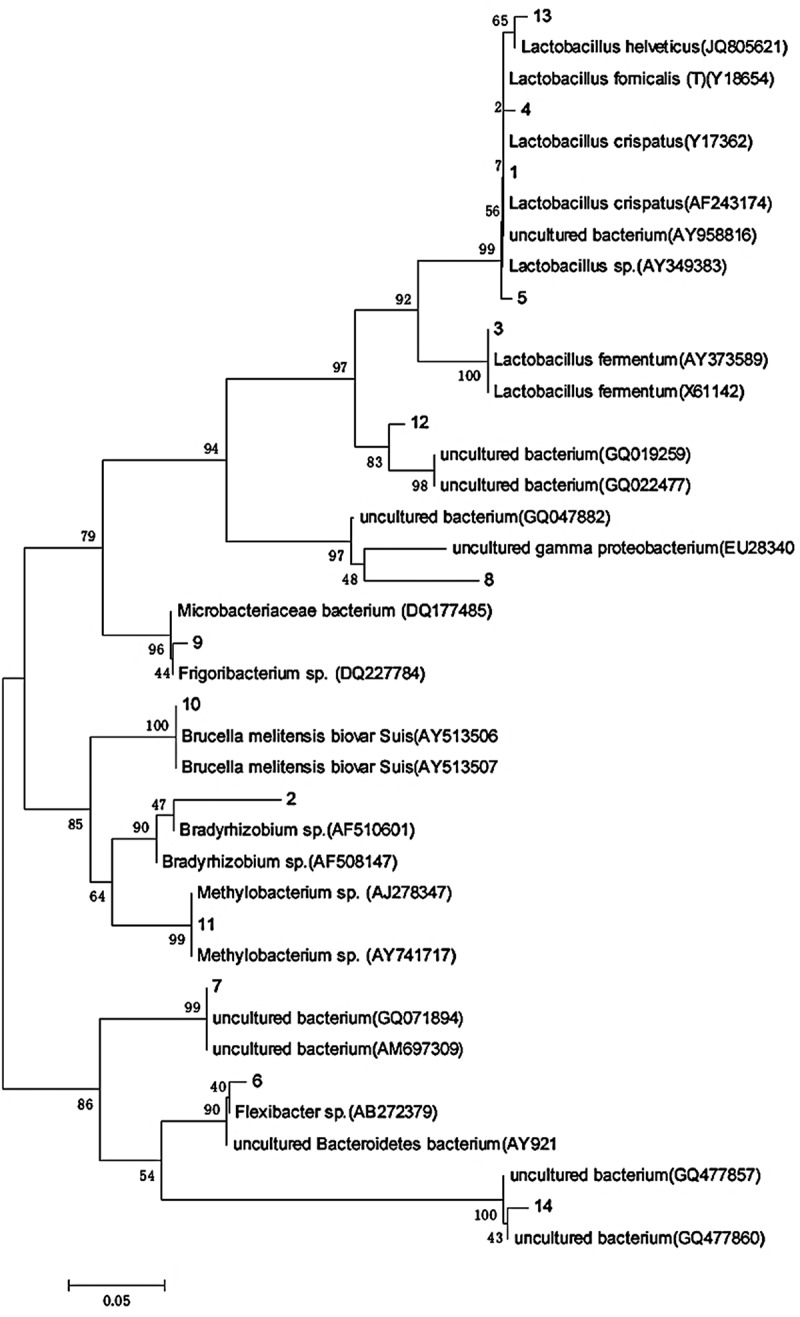
DGGE 16S rDNA phylogenetic tree

The phylogenetic tree was constructed by using the adjacency method (Figure 8). The length of the fragments ranged from 169bp to 194bp, which belonged to *Proteobacteria, Bacteroides, Firmicutes, Actinobacteria* and *Candidatus saccharicteria*. At the genus level, it is divided into 13 genera, most of which are nonculturable bacteria. The strong signal band was *Chitinophaga, Seiminibacterium* and *Cloacibacterium. Chitinophaga, Lactobacillus, Bradyrhizobium, Methylobacterium* and *Staphylococus*. The strong signal bands 5, 6, 1, 2, 11, and 12 belong to the following genera: *Chitinophaga, Semiminiature* and *Cloacibacterium, Chitinophaga, Lactobacillus, Bradyrizobium, Methylbacterium* and *Staphylococcus*, respectively. Studies have shown that *Flexicharacter sp*. and *Bradyrhizobium sp*. and *Methylbacterium sp*. are the dominant species in soil and water environment [23,24]. Therefore, the bacterial community composition in this study can not only reflect the specific environmental conditions, but also has certain particularity.

The air bacteria in Xuzhou city are mainly distributed in *Proteobacteria, Bacteroidetes* and *Pachytene*, indicating that these bacteria are more suitable for living in the atmospheric environment, which is consistent with the research results of domestic and foreign scholars who also adopt the molecular biological method [14,15,18,25,29]. Proteus bacteria are Gram-negative bacteria, widely distributed, and have a strong resistance to low temperature and radiation. The dominant bacteria in this group of samples is *Flexicharacter sp*., and several bacteria in this genus are opportunistic pathogens, which can cause infection of plant roots or animal wounds and cause various kinds of inflammation. There are also *Acinetobacter* species in this group of samples. They are opportunistic pathogenic bacteria from water and widely distributed in nature. Many kinds of bacteria in this genus are resistant to antibiotics. The infection caused by this genus of bacteria has increased in recent ten years. In addition, *Candidatus saccharacteria* in the sample is also a kind of opportunistic pathogen, which is harmful to children, immunosuppressive or immunodeficient patients and even the normal people. Air microorganism with different particle size can deposit in different parts, such as large particle size particles can be trapped in the upper respiratory tract, while small particle size particles can enter the lung through the upper respiratory tract, so air microorganism with different particle size has different inhalation risks [31]. The bacterial community structure in the fine particle size of the sample is more abundant than that in the coarse particle size. When haze occurs, the concentration of all kinds of pathogens in the fine particle size will increase, and the pathogens will also appear in the fine particle size.

## Future research perspectives

Microbes in the air are closely related to human life. Therefore, it is of great significance to study and monitor the concentration, species, distribution, and variation of airborne microorganisms.

Many researchers have conducted research on microorganisms in the air, but the level of research is generally low. Most researchers focus on the health risks of contamination of various chemical components, and few studies on the toxicity and harm of microbial populations have been reported. Future research perspectives of this work are the impact of microbial components in particulate matter on health risks, and the interaction between microorganisms and other major pollutants (SO_2_, NO_x_ and O_3_) in polluted air.

## Conclusion

In this study, PM_10_ and PM_2.5_ pollution levels and their changes in different functional areas and seasons of typical industrial cities in Northern Jiangsu were investigated. The proportion of fine particles (PM_2.5_) in PM_10_ is larger than that of coarse particles (about 58%). The physicochemical properties of PM_2.5_ were analyzed by SEM and EDS. Denaturing gradient gel electrophoresis (DGGE) was used to study the distribution characteristics of bacterial community structure on atmospheric particulates (TSP, PM_2.5_ and PM_10)_ in different functional areas of Xuzhou city in the winter haze. Based on the analysis of microbial diversity and community similarity in different particle sizes, it was found that the microbial populations in Xuzhou city atmospheric particles were mainly divided into three groups: *Proteobacteria, Bacteroidetes* and *Pachytenella*. The community structure of bacteria in fine particle size was more abundant than that in coarse particle size. When haze occurs, the concentration of all kinds of pathogens in fine particle size will increase, and the pathogens also will appear in fine particle size.

## Supplementary Material

Supplemental Material

## Data Availability

The datasets used and/or analyzed during the current study are available from the corresponding author on reasonable request.

## Authors’ information

**Affiliation**

School of Chemistry and Materials Science, Jiangsu Key Laboratory of Green Synthetic Chemistry for Functional Materials, Jiangsu Normal University, Xuzhou 221,116, P.R. China

Xingcheng Yuan, Guangchao Li, Weihua Yang, Dan Li
