# Supplementary figures and images for: Distribution characteristics of microbial community structure in atmospheric particulates of the typical industrial city in Jiangsu province, China
Source: Bioengineered. 2021 Feb 10;12(1):615–26. doi: 10.1080/21655979.2021.1885223 (PMC8806265; doi:10.1080/21655979.2021.1885223)

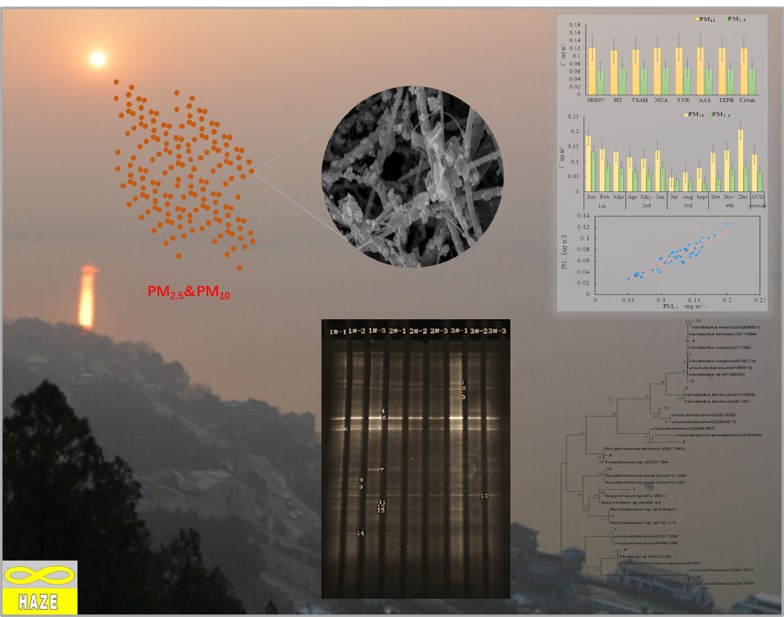

Supplement: Supplemental Material [file KBIE_A_1885223_SM8465.jpg]
